# Predicting intravitreal treatment response using ultrawide-field angiographic biomarkers in diabetic retinopathy^☆^

**DOI:** 10.1016/j.aopr.2025.10.003

**Published:** 2025-10-25

**Authors:** Karthik Reddy, Callie Deng, Boonkit Purt, Yue Liang, Nikhil Bommakanti, Gina Yu, Julie Rosenthal, Yannis M. Paulus

**Affiliations:** a University of Michigan Medical School, Ann Arbor, MI, USA; b Department of Ophthalmology and Visual Sciences, W. K. Kellogg Eye Center, University of Michigan, Ann Arbor, MI, USA; c Wills Eye Hospital, Sidney Kimmel Medical College, Philadelphia, PA, USA; d Department of Ophthalmology, Byers Eye Institute, Stanford University, Palo Alto, CA, USA; e Department of Ophthalmology, Wilmer Eye Institute, Johns Hopkins University, Baltimore, MD, USA; f Department of Biomedical Engineering, Johns Hopkins University, Baltimore, MD, USA

**Keywords:** Diabetic retinopathy, Ultra-widefield fluorescein angiography, Anti-VEGF, Steroid, Non-perfusion area, Neovascularization, Foveal avascular zone

## Abstract

**Background:**

Diabetic retinopathy (DR) is a sight-threatening retinal disease with pathological mediation by vascular endothelial growth factor (VEGF). Intravitreal anti-VEGF injections are commonly used to manage DR. Ultra-wide-field fluorescein angiography (UWF-FA) can assess DR severity and characterize the amount of non-perfused retina (non-perfusion area, NP), neovascularization (neovascular area, NV), and foveal avascular zone (FAZ). However, the association between anti-VEGF treatment and NP, NV, and FAZ characterized by UWF-FA is not well established.

**Methods:**

A retrospective, single-center cohort study involved eyes of patients with Type 1 or 2 diabetes mellitus with at least one UWF-FA image. The area of the FAZ, NP area, and NV area of UWF-FA images was calculated. Stepwise multivariate logistic regression was used to identify patient and eye-level factors that were significant predictors of FAZ, NP, and NV. Causal model analyses estimated intravitreal treatment effects on FAZ, NP, and NV over time.

**Results:**

The study included 705 eyes from patients with a mean (SD) age of 59.2 (13.2) years, and 56.3% were male. Eyes were treated with a mean (SD) of 5.6 (7.7) anti-VEGF and 0.63 (1.94) intravitreal steroid injections. Each incremental increase in Early Treatment Diabetic Retinopathy Study (ETDRS) severity of DR on initial presentation by clinical exam was associated with a 12.86 mm^2^ increase in total NP and a 0.73 mm^2^ increase in NV area. Anti-VEGF and steroid treatment had no significant impact on FAZ area. The presence of intravitreal steroid treatment was associated with an estimated decrease of −0.909 mm^2^ in total NV area (*P* = 0.009). Each additional anti-VEGF injection decreased NV area by −0.239 mm^2^ (*P* = 0.031). Any steroid use also led to an estimated −10.79 mm^2^ decrease in NP (*P* = 0.041). Each additional anti-VEGF injection was predictive of a −2.54 mm^2^ decrease in NP (*P* = 0.019). Each additional steroid injection was predictive of a −13.68 mm^2^ decrease in NP (*P* = 0.001).

**Conclusions:**

Intravitreal treatment was significantly associated with reduced NV and NP on UWF-FA. Intravitreal treatments were not predictive of FAZ changes. These findings suggest total retinal NV and NP areas may provide utility as UWF-FA biomarkers for assessing intravitreal treatment response.

## 1 Introduction

Diabetic retinopathy (DR) is a leading cause of vision loss, affecting an estimated 28.5% of diabetic adults in the US, with disproportionate impacts in marginalized communities.^1^^,^^2^ Neovascularization (NV) and non-perfusion (NP) are pathologic features related to the vascular component of diabetic retinopathy.^3^ Neovascularization is the formation of new blood vessels in response to ischemia, primarily driven by vascular endothelial growth factor (VEGF) expression. Proliferative DR, a subtype of DR, is associated with neovascularization and may lead to further complications such as hemorrhage, detachment, and glaucoma.^4^ NP is associated with the closure of the retinal microvasculature, leading to ischemic regions. This is associated with the release of pro-angiogenic factors such as VEGF, and NP is predictive of progression to the proliferative subtype, worsening vision, and increased NV.^3^^,^^5^ These findings are especially notable on ultrawide-field fluorescein angiography (UWF-FA), allowing additional peripheral visualization.^6^

Current strategies for managing diabetic retinopathy include intravitreal injections, specifically anti-VEGF and corticosteroid injections.^7^ However, the effect of these treatment modalities on DR ocular pathology, as characterized by UWF-FA, is mixed. Overall, some studies suggest that reductions in peripheral ischemia and signs of reperfusion occur with anti-VEGF injections; however, other studies suggest that this may not be the case, even with extended follow-up periods of 3–12 months.^8^ Much of the current evidence utilizes case reports or small cohorts of eyes with limited follow-up and injection counts, which may contribute to varying outcomes between cohorts.^8^, ^9^, ^10^, ^11^, ^12^, ^13^, ^14^, ^15^ While corticosteroids are known to reduce inflammation and vascular permeability, leading to decreased macular edema, their specific effects on peripheral retinal nonperfusion or ischemia, as visualized through UWF-FA, have not been extensively studied.^16^ One study using UWF-FA for eyes treated with dexamethasone implants revealed sustained improvement in retinal perfusion; however, this was limited by the small sample size.^17^

The mixed evidence limits the utility of UWF-FA in assessing DR pathology's response to intravitreal treatment, despite its novel ability to visualize peripheral changes that more accurately capture relevant pathologic changes compared to current standards in retinal imaging.^18^^,^^19^ UWF-FA may also provide insights into unrecognized features of DR and could aid in better prognostication and classification during treatment. Thus, further work is needed to better characterize the retinal characteristics associated with the treatment of DR using UWF-FA, informing future adoption of UWF-FA in prognostication, classification, and treatment monitoring for DR.^20^, ^21^, ^22^

This study aims to evaluate the associations between retinal perfusion, NV, and the foveal avascular zone (FAZ), as quantified through UWF-FA, and the administration of intravitreal anti-VEGF and corticosteroid injections in eyes affected by DR. By analyzing quantitative metrics, such as NP areas, NV extent, and FAZ dimensions, across the entire retina, including peripheral regions, the study aims to determine how these imaging biomarkers correlate with the level of treatment and disease progression in DR.

## 2 Methods

### 2.1 Study design and participants

This retrospective, single-center cohort study investigated the relationship between the number of intravitreal anti-VEGF injections and changes in retinal perfusion and neovascularization as observed in UWF-FA. Data were collected from patients diagnosed with type 1 or type 2 diabetes mellitus (DM) who underwent UWF-FA imaging at the University of Michigan Kellogg Eye Center between January 2009 and May 2018. The study received approval from the Institutional Review Board (HUM00120509) and adhered to the tenets of the Declaration of Helsinki. Given the study's retrospective nature, a consent waiver was obtained.

### 2.2 Inclusion and exclusion criteria

Patients were eligible for inclusion if they were at least 18 years old, had a confirmed diagnosis of type 1 or type 2 diabetes mellitus, and had undergone UWF-FA imaging sessions, with the final FA occurring after treatment with intravitreal anti-VEGF injections. Patients remained included if they received intravitreal steroid treatment with or without anti-VEGF treatment; however, not all patients received steroid treatment. Patients were excluded if they had undergone prior pan-retinal photocoagulation , had poor image quality that prevented accurate segmentation of NP and NV areas, or had significant media opacities such as cataracts or vitreous hemorrhage at the time of UWF-FA acquisition.

### 2.3 Image collection and processing

**Fig. 1:**
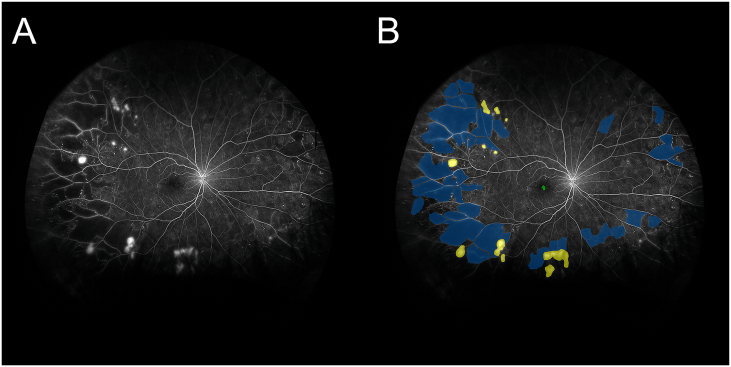
(A) Example of ultrawide-field FA and (B) UWF-FA images overlayed with segmentations of areas of non-perfusion (blue), neovascularization (yellow), and foveal avascular zone (green).

UWF-FA images were obtained using the Optos 200Tx or California (Optos PLC, Scotland, UK) scanning laser ophthalmoscopes. To correct peripheral distortion, images were mapped onto a standardized curved surface model with a nominal eye diameter of 24 mm using proprietary Optos software. Image segmentation occurred on early arteriovenous-phase fluorescein angiography (FA) images (20–45 s post-injection), defined as the first frame showing laminar venous filling with minimal leakage. If the predefined frame was non-diagnostic (e.g., motion artifact), the nearest adjacent frame within the same phase was used. Image segmentation was performed using ITK-SNAP, an open-source tool that enables semi-automated active contour segmentation. Four trained graders, masked to treatment history, manually delineated and quantified the FAZ, NP areas, and NV areas. NP and NV areas were further categorized based on anatomical location, including the posterior pole (≤3.00 mm from FAZ), mid-periphery (3.00–10.00 mm from FAZ), and far periphery (10.00–15.00 mm from FAZ) as demonstrated in Fig. 1. The surface areas of these regions across the retina were summed and quantified in square millimeters. All segmentation results were reviewed for consistency and accuracy by a senior retinal specialist.

Clinical and demographic data were collected through electronic medical record review, including patient age, sex, diabetes type, hemoglobin A1c (hemoglobin A1c) levels, and DR severity as classified by the Early Treatment Diabetic Retinopathy Study (ETDRS) scale.^19^ Detailed treatment history was recorded, focusing on the total number of anti-VEGF injections at final FA and the presence of diabetic macular edema (DME) or vitreous hemorrhage at baseline or during follow-up.

### 2.4 Statistical analyses

The primary objective of this study was to assess the relationship between the number of anti-VEGF injections and the final FA biomarkers, particularly total NP and NV areas. Univariate and multivariate regression analyses were conducted to assess this and identify independent predictors of final NP and NV areas. All statistical analyses were performed using R (version 4.3.3), and statistical significance was defined as *P* < 0.05.

Linear mixed models were used to evaluate the association of anti-VEGF injection count on final NP and NV areas while adjusting for baseline severity, diabetes type, and other relevant clinical factors. Covariates included age, sex, diabetes type, presence of DME, development of vitreous hemmorhage, and time between FA sessions. To mitigate selection bias, inverse probability weighting was applied using propensity score adjustments to balance covariates between eyes that received different numbers of injections. The association of anti-VEGF treatment on final FA biomarkers was estimated using weighted regression models. Stepwise multivariate logistic regression was used to identify patient and eye-level factors that are statistically significant predictors of total FAZ, total NP, and total NV.

## 3 Results

### 3.1 Cohort characteristics

The study included 705 eyes with a mean age of 59.2 ± 13.2 years, and 56.3% were male. The mean HbA1c was 7.95% ± 1.98%, and the mean DR severity score at presentation was 2.78 ± 1.57. DME was present in 45.4% of eyes, while all eyes (100%) had no vitreous hemorrhage (VH) at baseline. Throughout treatment, eyes received a mean of 5.59 ± 7.71 anti-VEGF injections, while steroid injections were less frequent (0.63 ± 1.94 per eye), as described in Table 1.

**Table 1:** Demographic characteristics of the study group.

Demographics	Overall
n	705
Age (mean (SD))	59.23 (13.19)
Sex = 1 (%)	397 (56.3)
Race (%)	
White	467 (66.2)
Black	159 (22.6)
Asian	27 (3.8)
Hispanic/Black	2 (0.3)
Hispanic	8 (1.1)
Hispanic/Other	11 (1.6)
Other (Hawaiian, Native American, Pacific Islander)	11 (1.6)
NA	20 (2.8)
Last BMI (kg/mÂ^2^ (mean (SD))	32.64 (8.59)
Last HbA1c (mean (SD))	7.95 (1.98)
logMAR VA (mean (SD))	0.34 (0.32)
DR Severity on Presentation by Clinical Exam (mean (SD))	2.78 (1.57)
DME on Presentation by Clinical Exam = 1 (%)	320 (45.4)
VH on Presentation by Clinical Exam = 0 (%)	705 (100.0)
Anti-VEGF (mean (SD))	5.59 (7.71)
Steroid (mean (SD))	0.63 (1.94)

BMI, body mass indexHbA1c, hemoglobin A1C; logMAR VA, logarithm of the Minimum Angle of Resolution; DR, diabetic retinopathy; DME, diabetic macular edema; VH, vitreous hemorrhage; Anti-VEGF, anti vascular endothelia growth factor; SD, standard deviation.

### 3.2 Identification of covariates

Baseline measurements were calculated for all participants. Total FAZ had a mean (SD) of 0.584 mm^2^ (0.619 mm^2^), total NP area had a mean area of 72.213 mm^2^ (56.614 mm^2^), and total NV area had a mean of 1.003 mm^2^ (5.320 mm^2^). Among our cohort, DME on initial presentation, as determined on clinical examination, had low predictive value but was a significant predictor of subsequent total FAZ level (B = −0.05, *P* = 0.046). For total NP, sex (*P* = 0.035), DR severity on clinical exam (*P* < 0.0001), development of VH (*P* = 0.012), and number of anti-VEGF injections (*P* = 0.029) were significant predictors. However, steroid use was not significantly predictive of NP (*P* = 0.094). Age (*P* = 0.005), DR severity (*P* = 0.002), and development of DME (*P* < 0.001) were significant predictors of NV. These results and non-significant predictors are further described in Table 2.

**Table 2:** Factors predictive of total FAZ, NP, and NV.

	**Mean**	**SD**		
**Total FAZ**	0.584	0.619		
**Covariates**	**Estimate**	**Std. Error**	**Pr (>|*t*|)**	
Race	0.05	0.03	0.088	.
Type of DM	0.05	0.03	0.062	.
DME on Presentation	−0.05	0.02	0.046	∗

	**Mean**	**SD**		
**Total NP**	72.213	56.614		
**Covariates**	**Estimate**	**Std. Error**	**Pr (>|*t*|)**	
Sex	5.15	2.44	0.035	∗
DR Severity on Presentation	12.86	2.39	0.000	∗∗∗
Developed VH	4.67	1.85	0.012	∗
Use of Steroid	−3.55	2.12	0.094	.
Use of Anti-VEGF	5.16	2.36	0.029	∗

	**Mean**	**SD**		
**Total NV**	1.003	5.320		
**Covariates**	**Estimate**	**Std. Error**	**Pr (>|*t*|)**	
Age	−0.66	0.23	0.005	∗∗
DR Severity on Presentation	0.73	0.23	0.002	∗∗
Developed DME	−0.77	0.21	0.000	∗∗∗

∗ Statistically significant *p*-value <0.05; ∗∗ significant to *p*-value <0.01; ∗∗∗ significant to *p*-value <0.001.

FAZ, foveal avascular zone area; NP, nonperfusion area; NV, neovascularization zone area; DM, diabetes mellitus; DME, diabetic macular edema; VH, vitreous hemmorhage; DR, diabetic retinopathy; SD, standard deviation.

### 3.3 Analysis of treatment effects on FAZ, total NP, and total NV

After confounding variables were identified on the stepwise linear regression model, the causal model results are displayed below in Table 3. In our study, no variables had causal implications on total FAZ that reached statistical significance.

**Table 3:** Causation analysis of treatment effects on total FAZ, NP, and NV.

	**Mean**	**SD**		
**Total FAZ**	0.584	0.619		
**Covariates**	**Estimate**	**Std. Error**	**Pr (>|*W*|)**	
Use of Anti-VEGF	0.049	0.053	0.362	
Use of Steroids	−0.007	0.048	0.890	
Number of Anti-VEGF Injections	−0.002	0.008	0.840	
Number of Steroid Injections	−0.021	0.028	0.460	

	**Mean**	**SD**		
**Total NP**	72.213	56.614		
**Covariates**	**Estimate**	**Std. Error**	**Pr (>|*W*|)**	
Use of Anti-VEGF	2.130	6.890	0.760	
Use of Steroids	−10.790	5.280	0.041	∗
Number of Anti-VEGF Injections	−2.540	1.079	0.019	∗
Number of Steroid Injections	−13.680	4.080	0.001	∗∗∗

	**Mean**	**SD**		
**Total NV**	1.003	5.320		
**Covariates**	**Estimate**	**Std. Error**	**Pr (>|*W*|)**	
Use of Anti-VEGF	−0.662	0.482	0.170	
Use of Steroids	−0.909	0.346	0.009	∗∗
Number of Anti-VEGF Injections	−0.239	0.111	0.031	∗
Number of Steroid Injections	−0.677	0.348	0.052	.

∗ Statistically significant *p*-value <0.05; ∗∗ significant to *p*-value <0.01; ∗∗∗ significant to *p*-value <0.001.

FAZ, foveal avascular zone area; NP, nonperfusion area; NV, neovascularization zone area; Anti-VEGF, anti-vascular endothelial growth factor; SD, standard deviation.

The presence of any intravitreal steroid treatment compared to no steroid use was associated with a −10.79 mm^2^ decrease (SE = 5.28, *P* = 0.041) in total NP. For every additional steroid injection, there was a −13.680 mm^2^ decrease in total NP (SE = 4.080, *P* = 0.001). The presence or absence of anti-VEGF use was not a significant factor in determining final total NP (B = 2.130 mm^2^, SE = 6.890, *P* = 0.760). However, every anti-VEGF injection led to a significant estimated decrease of −2.540 mm^2^ (SE = 1.079, *P* = 0.019).

The average total NV area of the cohort was 1.003 mm^2^ (SD = 5.320). With total NV, the effect of steroid use had an estimated decrease of −0.909 mm^2^ (SE = 0.346, *P* = 0.009). Each additional steroid injection had an estimated −0.677 mm^2^ decrease in total NV (SE = 0.111, *P* = 0.031). The presence of anti-VEGF use did not have a significant effect on total NV (B = −0.662 mm^2^, SE = 0.482, *P* = 0.170). Each additional anti-VEGF injection decreased total NV by −0.239 mm^2^ (SE = 0.111, *P* = 0.031). These results are further represented in Table 3.

## 4 Discussion

This study identifies relevant patient characteristics that predict neovascularization, non-perfusion, and foveal avascular zone size in the largest known retrospective cohort of eyes with DR. Additionally, this study examines the relationship between intravitreal treatment (anti-VEGF injections and intravitreal steroid injections) and key characteristics of DR pathology on UWF-FA.

Our results suggest that DME on presentation was related to a decreased FAZ among a cross-sectional cohort of eyes at a final timepoint. This concurs with a recent study that similarly corroborated a smaller FAZ's relationship with predicting DME.^23^ Similarly, sex, DR severity, vitreous hemmorhage (VH) development, and need for anti-VEGF treatment were also predictive of the area of non-perfusion, while increasing age, DR severity, and development of DME were predictive of neovascularization.

A previous study, utilizing a subset of our cohort (363 patients), identified male sex as a predictor of both NV and NP. However, our work only found sex predictive of NP.^24^ Jiang et al. similarly identified that macular leakage and presence of DME on UWF-FA were predictive of the need for treatment. However, no past study has identified the predictive value of these patient characteristics on quantitative UWF-FA biomarkers.^25^ Overall, these findings indicate that specific patient and disease characteristics, such as sex, age, DR severity, VH, need for anti-VEGF treatment, and DME, can predict the extent of retinal ischemia and neovascularization, potentially enabling personalized surveillance and timely intervention in diabetic retinopathy.

We also used inverse propensity weighting to mitigate confounding and infer the predictive value of anti-VEGF and steroid treatment on FA biomarkers. Significant relationships existed between treatment type and volume of quantitative biomarkers of DR pathology. While no treatment characteristic had significant predictive value for FAZ, treatment type and volume of both steroid and anti-VEGF injections were predictive of NP and NV.

There are many potential reasons for the lack of predictive value for FAZ. In eyes with DR, FAZ differs from peripheral NP and NV. The FAZ is a small, highly individualized region at the center of the macula whose size is determined mainly by baseline anatomy and undergoes only subtle change over time.^26^^,^^27^ Longitudinal studies also show that the FAZ area often remains statistically unchanged after treatment, further limiting predictive power.^28^ Because the FAZ area has high inter-individual variability, is less sensitive to macular capillary remodeling, and carries substantial measurement noise, treatment characteristics (type or volume of injections) fail to predict its size.

However, the predictive value of treatment volume and type to NP and NV has significant support in the literature. NP and NV are dynamic, VEGF-driven processes in the peripheral retina that respond directly to anti-VEGF and steroid therapies.^29^^,^^30^ Our study demonstrated a large effect size for steroid-based intravitreal treatment modalities, with the use of steroids predictive of a 10.790 mm^2^ decrease in NP and 0.909 mm^2^ in NV. Additionally, each additional steroid injection resulted in a −13.680 mm^2^ decrease in NP. This is broadly in line with the limited literature on steroid efficacy, with several trials demonstrating corticosteroid suppression of inflammatory and VEGF pathways and varying degrees of attenuation of ischemic and neovascular processes, such as Borrelli et al. demonstrating an approximate 14.5% reduction in ischemic index using intravitreal steroid implants.^17^^,^^31^^,^^32^

While the relationship between UWF-FA biomarkers and anti-VEGF has been mixed in the literature,^8^, ^9^, ^10^, ^11^, ^12^, ^13^, ^14^, ^15^ our study demonstrated a significant association between additional anti-VEGF injection counts and reduced both NP and NV; however, the use of anti-VEGF was not predictive. This is likely due to the need for a continuous, intensive anti-VEGF regimen to achieve and measure significant reductions in NP and NV on UWF-FA.^33^^,^^34^ In contrast, a binary variable of ''any use'' fails to distinguish between minimally treated eyes and those receiving complete, sustained therapy—hence its lack of predictive power in our analysis.

The strengths of this study included being the largest known retrospective cohort of DR eyes who had undergone UWF-FA, which enabled the identification of previously unidentified predictors of pertinent biomarkers and associated treatment response. There were several limitations of this study. Patients in this study received both anti-VEGF and steroid injections. We also employed multivariable regression and causal inference analyses to account for potential confounders, including the natural progression of the disease and the relationship between DR severity and treatment frequency. Including several parameters to establish the most parsimonious model may result in overfitting. Together, these may limit the generalizability of these findings.

Additionally, some patients who have received care, including intravitreal treatment, outside of Kellogg Eye Center may limit the accuracy of our findings due to variations in treatment counts. Quantification of NP, NV, and FAZ is susceptible to variability from image quality, acquisition parameters, device differences, segmentation or threshold choices, and inter-grader interpretation. This may decrease reliability across visits and graders. We did not perform formal repeatability or reproducibility assessments; therefore, the metrics should be interpreted with caution. However, a senior retinal specialist was available for adjudication and reviewed the measurements for consistency.

A future investigation, such as a prospective study, would be beneficial in addressing these concerns. Additionally, further work should be done to establish the clinical utility of UWF-FA in treatment monitoring for DR and to assess the treatment effects of intravitreal steroids on peripheral retinal changes in DR using UWF-FA. Lastly, this study examined total changes in the retina during treatment. Still, future studies may benefit from assessing regional differences across macular and peripheral locations in association with treatment-related effects.

## 5 Conclusions

Intravitreal treatment had significant associations with reduced NV and NP on UWF-FA. However, neither steroids nor anti-VEGF injection counts were predictive of changes in the foveal avascular zone. These findings suggest that total retinal NV and NP, as characterized on UWF-FA, may have the potential to serve as biomarkers for assessing intravitreal treatment response. This has immense potential for long-term disease monitoring in diabetic retinopathy by utilizing a greater degree of the retina to capture responses to current treatment strategies, and can inform future clinical management and research.

## Study approval

The authors confirm that any aspect of the work covered in this manuscript that involved human patients or animals was conducted with the ethical approval of all relevant bodies and the study was performed in accordance with the Declaration of Helsinki, and the protocol was approved by the Ethics Committee of the Institutional Review Board (IRB) (approval number: HUM00120509).

## Author contributions

Karthik Reddy: conceptualization, investigation, writing – original draft, writing – reviewing & editing, visualization, data curation; Callie Deng: writing – reviewing & editing, visualization, data curation, methodology; Boonkit Purt: conceptualization, investigation, writing – reviewing & editing; Yue Liang: software, formal analysis, data curation; Nikhil Bommakanti: conceptualization, methodology, investigation, data curation, writing – review & editing, supervision; Gina Yu: data curation, methodology, investigation; Julie Rosenthal: supervision, investigation, writing – review & editing; Yannis Paulus: conceptualization, methodology, investigation, resources, data curation, writing – review & editing, supervision, project administration, funding acquisition.

## Funding

This work was supported by the NIH 1R01EY034325 and 1R01EY033000 (YMP), University of Michigan MTRAC-FFMI Kickstart, Fight for Sight- International Retinal Research Foundation (YMP: FFSGIA16002), VitreoRetinal Surgery Foundation (NB), Alcon Research Institute Young Investigator Grant (YMP), Helmut F. Stern Career Development Professorship in Ophthalmology and Visual Sciences (YMP), Fight for Sight International Retinal Research Foundation (YMP), and unrestricted departmental support from Research to Prevent Blindness (YMP).

## Declaration of competing interest

The authors declare that they have no known competing financial interests or personal relationships that could have appeared to influence the work reported in this paper.

## Abbreviations

DR: Diabetic Retinopathy
UWF-FA: ultra-widefield fluorescein angiography
FAZ: foveal avascular zone
NP: non-perfusion area
NV: neovascularization area
DM: diabetes mellitus
DME: diabetic macular edema
ETDRS: Early Treatment Diabetic Retinopathy Study
VH: vitreous hemmorhage
SD: standard deviation
VEGF: vascular endothelial growth factor
